# A set of regulatory genes co-expressed in embryonic human brain is implicated in disrupted speech development

**DOI:** 10.1038/s41380-018-0020-x

**Published:** 2018-02-20

**Authors:** Else Eising, Amaia Carrion-Castillo, Arianna Vino, Edythe A. Strand, Kathy J. Jakielski, Thomas S. Scerri, Michael S. Hildebrand, Richard Webster, Alan Ma, Bernard Mazoyer, Clyde Francks, Melanie Bahlo, Ingrid E. Scheffer, Angela T. Morgan, Lawrence D. Shriberg, Simon E. Fisher

**Affiliations:** 1 Language and Genetics Department, Max Planck Institute for Psycholinguistics, Nijmegen, 6525 XD The Netherlands; 2 Department of Neurology, Mayo Clinic, Rochester, MN 55905 USA; 3 Department of Communication Sciences and Disorders, Augustana College, Rock Island, IL 61201 USA; 4 Population Health and Immunity Division, Walter and Eliza Hall Institute of Medical Research, Melbourne, 3052 Australia; 5 Department of Medical Biology, University of Melbourne, Melbourne, 3010 Australia; 6 Faculty of Medicine, Dentistry and Health Sciences, University of Melbourne, Melbourne, 3010 Australia; 7 Department of Neurology and Neurosurgery, Children’s Hospital Westmead, Sydney, NSW Australia; 8 Department of Clinical Genetics, Children’s Hospital Westmead, Sydney, NSW Australia; 9 University of Bordeaux, IMN, UMR 5293, Bordeaux, France; 10 Donders Institute for Brain, Cognition and Behaviour, Radboud University, Nijmegen, 6500 HE The Netherlands; 11 Austin Health and Royal Children’s Hospital, Melbourne, 3052 Australia; 12 Neuroscience of Speech, Murdoch Childrens Research Institute, Melbourne, 3052 Australia; 13 Waisman Center, University of Wisconsin-Madison, Madison, WI 53705 USA

**Keywords:** Genetics, Neuroscience

## Abstract

Genetic investigations of people with impaired development of spoken language provide windows into key aspects of human biology. Over 15 years after *FOXP2* was identified, most speech and language impairments remain unexplained at the molecular level. We sequenced whole genomes of nineteen unrelated individuals diagnosed with childhood apraxia of speech, a rare disorder enriched for causative mutations of large effect. Where DNA was available from unaffected parents, we discovered de novo mutations, implicating genes, including *CHD3*, *SETD1A* and *WDR5*. In other probands, we identified novel loss-of-function variants affecting *KAT6A*, *SETBP1*, *ZFHX4*, *TNRC6B* and *MKL2*, regulatory genes with links to neurodevelopment. Several of the new candidates interact with each other or with known speech-related genes. Moreover, they show significant clustering within a single co-expression module of genes highly expressed during early human brain development. This study highlights gene regulatory pathways in the developing brain that may contribute to acquisition of proficient speech.

## Introduction

The capacity to acquire complex spoken language appears to be unique to humans [1]. The majority of children, when exposed to linguistic input in their environment, develop skills to understand others, to convert thoughts into spoken utterances and to produce intelligible speech. Remarkably, this sophisticated suite of abilities emerges rapidly within the first few years of life without the need for formal teaching or special effort. Several complementary lines of biological research suggest that there are strong genetic underpinnings for such skills, ranging from evidence of significant heritability from twin and family studies to observations of gene associations in molecular studies of relevant traits [2]. Identification of the responsible genes not only sheds novel light on the pathways underlying the disorders, but can also greatly enhance our fundamental knowledge about the neurobiological mechanisms enabling humans to acquire language [3].

Most cases of developmental language impairments are likely to involve genetic complexity, resulting from the inheritance of multiple risk factors with small individual effect sizes [2]. Nonetheless, it has been established that disorders of speech and language sometimes occur in monogenic form. One relevant disorder that may be enriched for damaging gene variants of large effect size is childhood apraxia of speech (CAS), also known as developmental verbal dyspraxia. CAS is a rare severe developmental disorder characterized by difficulties with automatically and accurately sequencing speech sounds into syllables, syllables into words, and words into sentences with the correct prosody [4]. In CAS, it is thought that impairments in the neural planning and/or programming of spatiotemporal parameters of movement sequences result in errors in speech sound production and prosody. Diagnostic features of CAS include inconsistency in the types of speech errors that are made, and greater problems as the complexity and length of the utterance increase.

In 2001, studies of a large multigenerational pedigree named the KE family, along with an unrelated case with a chromosomal translocation, led to the discovery that disrupting one copy of the *FOXP2* gene (on chromosome 7q31) is sufficient to cause CAS [5]. Multiple different cases of *FOXP2* disruption have since been identified, including missense and nonsense mutations, insertion/deletions and chromosomal rearrangements of various kinds; CAS is the most consistent phenotypic consequence in the affected people [2, 6]. More than 15 years after the identification of *FOXP2*, progress in identifying additional genetic risk factors has been slow, and mainly driven by studies of chromosomal rearrangements. For example, deletion of a ~600-kb region in human chromosomal band 16p11.2, encompassing >25 genes, significantly increases risk of CAS, amongst other phenotypic consequences [7]. The *BCL11A* and *ERC1* genes have been found to be disrupted by deletions at 2p16.1 and 12p13.33, respectively in children with CAS or with broader problems that also involve intellectual disability, motor difficulties, developmental problems (for *BCL11A* deletions) and psychiatric manifestations (for *ERC1* deletions) [8, 9]. Recently, point mutations that disrupt *BCL11A* function have been implicated in a neurodevelopmental syndrome that includes language delays, although a diagnosis of speech apraxia was not specifically reported in these cases [10]. Still, the majority of speech apraxia cases do not seem to carry causal mutations in *FOXP2*, *BCL11A* or *ERC1* [6, 11, 12].

The present study aimed to take advantage of cutting-edge genomic strategies to identify novel genes implicated in CAS, and thereby move the field significantly beyond *FOXP2*. In particular, we applied whole-genome sequencing (WGS) in 19 probands with a diagnosis of CAS, to identify single nucleotide variants (SNVs) and small insertions and deletions (indels) against a genome-wide background. Crucially, for half the WGS cohort of the present study (9 probands), we could also sequence the entire genomes of nuclear family members without CAS (both parents, and in one family a sibling as well), allowing us to directly pinpoint de novo variants, which are known to have an increased likelihood of being causal [13]. Moreover, given the established evidence that genes involved in speech and language disruptions cluster in related functional pathways [14, 15], we coupled our WGS findings to co-expression network analysis, identifying correlated expression patterns across developing human brain tissue samples. We further validated findings by comparison to WGS data from an independent set of healthy controls, analyzed using the same procedures as the CAS cohort. Our work uncovered a neural co-expression module of functionally-related genes, which brings together *ERC1* and *BCL11A* with newly implicated candidate genes in CAS susceptibility, several of which have also been connected with neurodevelopment through studies of other disorders.

## Patients and Methods

### Ethics

The research in this study was approved by the appropriate review boards: the Social and Behavioral Sciences Institutional Review Board of the University of Wisconsin-Madison [Protocol 2013-0438], the Augustana College Institutional Review Board, and the Mayo Clinic Institutional Review Board [Protocol PR08-002372] (primary CAS cohort); the Melbourne Human Research Ethics Committee [project 27053] (Australian case); and the Basse-Normandie local ethics committee [reference CPP-2006-16] (control data set).

### Participants

The primary data set comprised 19 probands who were ascertained based on a formal clinical diagnosis of CAS. Participants were recruited for a study of pediatric motor speech disorders at two collaborative sites, as described previously [11, 12]. All probands were evaluated using the Madison Speech Assessment Protocol and the Speech Disorders Classification System to identify and classify speakers’ speech status at assessment, including CAS (Supplementary Table 1) [16]. Medical genetic evaluations were not included in the assessment protocol. For 9 of the probands, 19 additional nuclear family members provided DNA samples for sequencing (i.e., unaffected parents for 9 children, plus one unaffected sibling). Blood samples were obtained from these 9 probands; blood and saliva samples (Oragene DNA OG-500 kit; DNA Genotek Inc., Kanata, Ontario, Canada) were obtained from the remaining 10 probands. All participants gave informed consent. The current study included 7 out of 10 probands from a previous WES-based study that primarily focused on known candidate genes, and 5 out of 12 probands previously studied using aCGH screening [11, 12]. However, neither of these prior studies included any analyses of parental/sibling DNA samples. Supplementary Table 2 summarizes the relationship with the two previously studied cohorts.

### Whole-genome sequencing and variant calling

Novogene (Hong Kong) performed WGS of the CAS cohort using Illumina’s HiSeq Xten technology, involving paired-end sequencing, with reads of 150 base pairs long and a library insert size of 350 base pairs. Clean raw reads made up 97% of the total reads and were mapped onto the human reference genome (hg19) using Burrows–Wheeler Aligner (BWA) [17]. BAM files were sorted using SAMtools [18] and PCR duplicated reads were marked using Picard. Re-alignment around indels (insertion/deletions) and base quality control recalibration was performed using Genome Analysis Toolkit Software (GATK v3.4) [19]. Genetic variants in the sequence data were called as recommended by GATK best practices. We performed Variant Quality Score Recalibration (VQSR) on the WGS data, excluding variants with a VQSR score over 99%.

Anonymized primary data are deposited at The Language Archive (TLA: https://corpus1.mpi.nl/), a public data archive hosted by the Max Planck Institute for Psycholinguistics. Data are stored at the TLA under the node ID: MPI2535402#, and accessible with a persistent identifier: https://hdl.handle.net/1839/BAC29352-0AF4-4A09-B946-F4AC4865A67E@view. Access can be granted upon request.

### Control data

WGS data of 22 unaffected unrelated individuals (12 female, 10 male), recruited as part of the BIL&GIN data set [20], were used as healthy controls to test for potential enrichment bias that might result from variant filtering procedures (see below). Informed consent was obtained from all participants. BGI (Hong Kong/Shenzhen) performed WGS on this data set using Illumina’s HiSeq Xten technology, involving paired-end sequencing with reads of 150 base pairs long (8 samples) or 90 base pairs long (14 samples). The same pipeline as that applied to the CAS cohort (updated to GATK v3.5, VEP v88 and Gemini v20.0) was used for WGS data alignment and variant calling, annotation and filtering.

### Structural variant calling

We used a combination of two algorithms for the detection of structural variants on the autosomes: BreakDancer [21] (v1.1.2) and BIC-seq2 [22] (v0.7.2), using standard settings for both. BreakDancer bases its detection of structural variants on abnormal alignment of read pairs. BIC-seq2 bases its detection of structural variants on read depth. Structural variants detected by both BreakDancer and BIC-seq2 with a maximum two-fold size difference and maximal distance of 10 kb between predicted start or end sites were considered.

### Variant annotation and filtering

Variant Effect Predictor [23] (v73) was used for annotation and Gemini [24] (v0.18.3) was used to select exonic variants in protein-coding genes from the data set. Variants located in known regions of genomic duplications, in regions with a sequencing depth below 10 reads and present in any of the unaffected nuclear family members were excluded. Variants were filtered further based on minor allele frequency (MAF), and expression of the gene in developing brain according to thresholds outlined below. In addition, in absence of information about de novo status, variants identified in the ten singleton probands for whom parental DNA was not available, were further filtered based on gene intolerance and predicted functional impact of the variant.

The threshold for MAF was 5 × 10^−4^ in 1000 genomes (1000G; phase 3) and the exome variant server (ESP; release ESP6500SI-V2) and a maximum tolerated allele count of 10 in the Exome Aggregation Consortium (ExAC; v0.3) database [25], based on a recent statistical framework that takes into account multiple factors, including disease prevalence, genetic heterogeneity, inheritance mode and penetrance [26].

Genes were considered intolerant based on a Residual Variation Intolerance Score (RVIS) <25 [27]. In addition, genes with loss-of-function (LoF) variants were considered if the probability of being LoF intolerant (pLi) was >0.9, and genes with missense variants were considered if the Z-score for missense constraint (MIS_Z) was >3 [25]. Missense variants with GERP++ >2, scaled CADD (v1.0) >15 and Polyphen and SIFT indicating a damaging effect were considered to have high impact.

As CAS is a neurodevelopmental disorder with early onset, only genes that are expressed in the developing human brain were included. Genes were considered expressed in the developing brain if transcripts were present in the developmental human RNA sequencing data set of Brainspan (http://www.brainspan.org/) in any brain tissue collected 8–24 weeks post conception, with >1 fragments per kilobase of exon per million reads mapped (FPKM). Established candidate genes for involvement in CAS (*FOXP2, BCL11A* and *ERC1*) and other speech/language-related developmental disorders (*ATP2C2, AUTS2*, *CMIP*, *CNTNAP2, CTNND2, DCDC2, DOCK4, DYX1C1, FOXP1, GRIN2A, KIAA0319, NFXL1, ROBO1, SETBP1* and *SRPX2*) [2] all passed this threshold.

### Variant interpretation

Phenotypes previously associated with similar variants occurring in the same gene were collected using searches in PubMed, the Online Mendelian Inheritance in Man (OMIM) database and the Human Gene Mutation Database (HGMD Professional, version 2016.3). Variants identified in the current WGS analysis were interpreted according to a five-tier system of classification for variants of Mendelian disorders into 1) pathogenic, 2) likely pathogenic, 3) uncertain significance, 4) likely benign and 5) benign variants [28].

All variants reported in the main manuscript were independently validated using Sanger sequencing.

### Co-expression network

Brainspan RNA-sequencing data were used to construct a co-expression network. All cerebral brain samples from fetal, neonatal and infancy periods (8 weeks post conception up to 12 months of age) were included. A total of 224 samples collected from multiple regions of 23 human brains with high RNA quality (RIN > 9), and 14,442 genes with high expression (≥1 FPKM in at least 2 samples), and variable expression between samples (>0 FPKM in at least 50% of samples and coefficient of variance >0.25) were included. Motivated by the prior literature on neural correlates of CAS, as well as knowledge of brain regions impacted by known CAS-related genes [29–34], the samples used for calculating the co-expression network included not only cortical regions but also subcortical structures (discussed further in “Results” section). Co-expression analysis was carried out using the weighted correlation network analysis (WGCNA) R package [35]. A signed weighted adjacency matrix was calculated from the log-transformed gene expression data, using biweight midcorrelations to calculate co-expression similarity, and a soft thresholding power of 31, which was transformed into a topological overlap matrix. Modules were detected using the cutreeDynamic function of the WGCNA package, with hybrid tree cutting, and a minimal cluster size of 200 genes (settings method = hybrid, deepSplit = 2, minClusterSize = 200). To summarize the expression pattern of the genes in a module, module Eigengenes are calculated as the first principal component.

### Gene set analysis

Enrichment of gene sets in co-expression modules was calculated using a two-sided Fisher exact test, followed by false discovery rate (FDR) correction for multiple-testing. For the enrichment analysis of the CAS candidate genes, we used a background set comprising the 2,143 genes of the genome that are intolerant to mutations and that are expressed in the developing brain, to compensate for the background enrichment pattern of intolerant genes in the network (Supplementary Fig. 1). In addition, we studied enrichment for lists of genes with de novo mutations in patients with intellectual disability (ID, *n* = 230), autism spectrum disorder (ASD, *n* = 2,760) and schizophrenia (*n* = 711) [36]. The enrichment of these gene lists has previously been studied in a co-expression network based only on cortical tissues [37].

To functionally interpret modules, gene ontology (GO) term enrichments were performed in DAVID 6.8 [38]. The Functional Classification Tool of DAVID was applied to group identified GO terms with low clustering stringency. Bonferroni-corrected geometrical mean *p* values for each group of GO terms were reported.

## Results

### Whole-genome sequencing was used to discover gene variants

Whole-genome sequencing (WGS) with on average 32.1 times sequencing depth was carried out for 38 DNA samples from 19 probands with CAS and 19 nuclear family members (Supplementary Tables 1 and 2). Before considering a genome-wide view, we used the data to assess potential contributions of three genes implicated in CAS in prior published work: *FOXP2*, *BCL11A* and *ERC1* [5, 8, 9], as well as the *FOXP2*-paralogue *FOXP1* that has been implicated in a broader speech-related neurodevelopmental phenotype [39]. Consistent with earlier limited studies of subsets of the present cohort, our analyses identified two rare missense variants: *FOXP1* (ENST00000318789, c.322A>G) p.I107T in proband 01 and *FOXP2* (ENST00000408937, c.1864A>C) p.N622H in proband 11. However, we found that the *FOXP1* variant was in fact inherited from the proband’s unaffected mother, casting doubt on a causal role in CAS. Moreover, recent molecular and cellular assays of both variants indicate that they do not impact on the function of the encoded proteins [39, 40]. We, therefore, classified both as variants of uncertain significance. Thus, prominent CAS-related risk genes from the literature do not appear to account for the disorder in this WGS data set.

### De novo variants disrupt CHD3, SETD1A and WDR5 in probands with CAS

We next took advantage of the trio/quartet design of part of our WGS sample to search for novel genes that might be implicated in CAS, the first systematic application of the de novo paradigm for assessing any speech disorder. Filtering on minor allele frequency of the variant in public databases (1000G, ESP and ExAC), and expression of the gene in the developing brain, yielded nine non-synonymous exonic de novo variants (Table 1; Supplementary Table 3). All nine variants were successfully validated and confirmed as de novo by Sanger sequencing. In addition, a 1.86 Mb deletion at 2q31.1 (chr2:172,788,173-174,646,059) in proband 06, detected previously in this proband using aCGH [11], was independently detected in our WGS analyses and now shown to represent a de novo structural variant. The deletion affects multiple contiguous genes (*HAT1, METAP1D, DLX1, DLX2, ITGA6, PDK1, RAPGEF4, ZAK* and *CDCA7*). Mutations of these deleted genes have not been implicated in a neurodevelopmental disorder in prior work, although common variation at *DLX1* and *DLX2* has been associated with increased risk of ASD [41]. Of note, all other structural variants and SNVs previously reported in cases 01–09 (Supplementary Table 2) [11, 12] were also found in unaffected nuclear family members of the probands, revealing that those variants are unlikely to be causal for CAS.

**Table 1:** De novo exonic protein-altering variants in trios with CAS

Proband	Chr	Base	Gene	Transcript	cDNA change	Protein change	Impact	RVIS^a^	pLi^a^	MIS_Z^a^	GERP^b^	CADD^c^	Polyphen^d^	Sift^e^	Classification
01	17	7806599	*CHD3*	ENST00000380358	c.3682C>T	p.R1228W	Missense	+	NA	+	–	+	PrD	D	Pathogenic
02	18	9221974	*ANKRD12*	ENST00000262126	c.920C>G	p.S307C	Missense	+	NA	–	+	+	PrD	D	VUS
	11	62594638	*STX5*	ENST00000294179	c.412A>G	p.I138V	Missense	−	NA	–	+	+	PrD	T	VUS
	19	607997	*HCN2*	ENST00000251287	c.1252C>G	p.L418V	Missense	+	NA	+	+	+	PosD	T	VUS
04	16	30976714	*SETD1A*	ENST00000262519	c.1652_1656dup	p.P553Wfs*110	Frameshift	+	+	NA	+	NA	NA	NA	Pathogenic
07	9	137017143	*WDR5*	ENST00000358625	c.623C>T	p.T208M	Missense	+	NA	+	+	+	PosD	D	Pathogenic
08	1	212792854	*ATF3*	ENST00000366981	c.503A>C	p.N168T	Missense	–	NA	–	+	+	B	D	VUS
	8	144944141	*EPPK1*	ENST00000525985	c.3281G>A	p.S1094N	Missense	NA	NA	–	+	–	B	D	VUS
	15	63943544	*HERC1*	ENST00000443617	c.10454G>A	p.S3485N	Missense	+	NA	+	+	+	B	T	VUS

Variants were classified according to recent guidelines [28]

*Chr* chromosome, *RVIS* residual variation intolerance score, *pLi* probability of being loss-of-function intolerant, *MIS_Z* Z-score for missense constraint, *GERP* genomic evolutionary rate profiling, CADD combined annotation dependent depletion, Polyphen polymorphism phenotyping, sift: sorting intolerant from tolerant, *NA* not available or not applicable, *VUS* variant of uncertain significance

^a^Plus-sign: score indicating intolerant gene, minus-sign: score indicating tolerant gene

^b^Plus-sign: score indicating conserved base, minus-sign: score indicating not conserved base

^c^Plus-sign: score indicating intolerated variant, minus-sign: score indicating tolerated variant

^d^PrD: probably damaging, PosD: possibly damaging, B: benign

^e^D: deleterious, T: tolerated

^a–e^Scores on which classification is based can be found in Supplementary Table 3

**Fig. 1:**
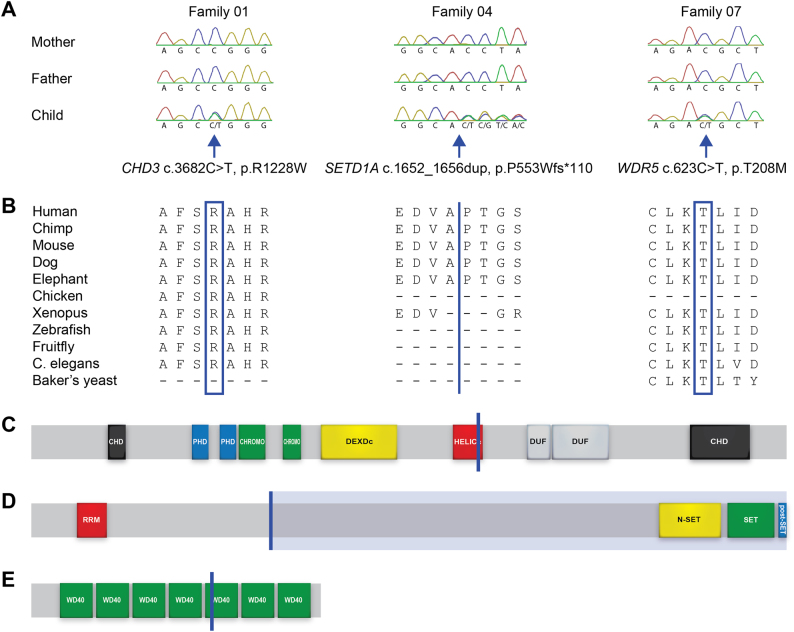
De novo pathogenic variants in *CHD3*, *SETD1A* and *WDR5*. **a** Sanger validation of de novo pathogenic variants in family 01, 04 and 07. **b** Conservation of the mutated amino acids across species. Blue boxes mark the mutated amino acid. The blue line indicates the site of the duplication. **c** Schematic representation of CHD3 (ENST00000380358) organization, with CHD C- and N-terminal domains in black, plant homeodomain (PHD) zinc finger domains in blue, chromatin organization modifier (CHROMO) domains in green, DEAD-like helicases superfamily (DEXDc) domain in yellow, a helicase superfamily c-terminal (HELICc) domain in red and two domains of unknown function (DUF) in gray. Blue line indicates site of the p.R1228W variant. **d** Schematic representation of SETD1A (ENST00000262519) protein, with an RNA recognition motif (RRM) in red, a complex proteins associated with Set1p (COMPASS) component N (N-SET) domain in yellow, a Su(var)3–9, Enhancer-of-zeste, Trithorax (SET) domain in green and a post-SET domain in blue. Blue line indicates site of the frameshift, and the blue shaded area indicates the deleted part of the protein. **e** Schematic representation of WDR5 (ENST00000358625), with WD40 repeats in green. Blue line indicates site of the p.T208M variant

In each of probands 01, 04 and 07, a single exonic de novo variant was identified (Fig. 1). Proband 01 carries a de novo missense variant (p.R1228W in ENST00000380358; p.R1169W in ENST00000330494) in a key functional domain of *CHD3*, a chromatin remodeling factor. In proband 04, a de novo loss-of-function (LoF) variant (p.V553Wfs*110) was found disturbing *SETD1A*, a histone methyltransferase. In proband 07, a de novo missense variant (p.T208M) was found in *WDR5*, within the WD40-repeat domain of the encoded protein. In each case, the respective variant was predicted to be pathogenic and there was strong independent evidence supporting causality from prior studies of the gene in question. Following our discovery of the *CHD3* variant in proband 01, de novo variants disrupting this gene have now been pinpointed in 34 other cases worldwide, with a variable neurodevelopmental disorder in which impaired speech and language is one of the phenotypic features (L. Snijders Blok, personal communication). In addition, LoF variants in *SETD1A* have been associated with neurodevelopmental disorders that include schizophrenia, ID and speech/language delays [42]. Lack of symptoms indicating developmental delay or a psychiatric disorder suggests that proband 04, who carries the *SETD1A* frameshift, has a mild form of *SETD1A*-associated disorder. Lastly, *WDR5* and *SETD1A* have close functional connections, since the proteins they encode belong to the same protein complex that confers histone-3 lysine-4 methylation [43]. For probands 02 and 08, three de novo variants were identified in each case (Table 1), all classified as variants of unknown significance.

### Loss-of-function variants in CAS implicate known neurodevelopmental genes

We went on to study the genomes of the 10 singleton probands for whom parental DNA was not available. On searching for variants in any of the nine genes already shown to carry de novo variants (Table 1), proband 15 had a missense variant in *HERC1* (c.2932-2933GA>TC, p.E978S). *HERC1* is associated with a recessive type of ID [44], but it is unknown whether heterozygous variants could lead to CAS.

Broadening to a genome-wide view, strict filtering criteria based on minor allele frequency, gene intolerance and expression of the gene in developing brain were applied to obtain only the most likely causal variants. A total of seven LoF variants in five probands were identified (Table 2). Interestingly, four of these LoF variants disrupted genes previously implicated in neurodevelopmental disorders: *KAT6A* (c.1599-56_1621del in proband 10), *SETBP1* (c.1781del, p.P594Lfs*36 in proband 13), *TNRC6B* (c.2040G>A, p.W680* in proband 15) and *ZFHX4* (c.3646-1G>A in proband 14) [45–48]. These four mutations were therefore considered pathogenic. LoF variants in *KAT6A* are associated with severe speech delay, ID, hypotonia and facial dysmorphism [45]. To our knowledge, proband 10 of the present study represents the first case of a *KAT6A* LoF variant that only yields a speech phenotype, in absence of global developmental delay. Recurrent de novo LoF mutations were recently identified in *TNRC6B* in children with ASD in a large cohort of over 2,500 families [47], while deletions of *ZFHX4* have been associated with ID and specific facial features [48]. The neurodevelopmental features of proband 13 (CAS, intellectual deficits and motor delay) overlap with the clinical picture associated with *SETBP1* haploinsufficiency in previous reports [46]. In this proband, as well as the frameshift in *SETBP1*, another two LoF variants were present (in *OPA1* and *RAP1GAP*), but considered of less significance for CAS, given prior literature on *SETBP1* dysfunction and speech/language phenotypes. Lastly, proband 18 carries a splice acceptor variant in *MKL2*. A recent large-scale targeted sequencing study of ~300 brain-related genes in ASD identified rare variants of *MKL2*, but the relevance of these for the disorder was uncertain [49]. Based on the functional consequence of the *MKL2* variant observed in proband 18 and the gene intolerance, we considered this variant as likely pathogenic.

**Table 2:** Rare loss-of-function variants in intolerant genes identified in ten singleton cases with CAS

Proband	Chr	Base	Gene	Transcript	cDNA change	Protein change	Impact	RVIS	pLI	Phenotype previously associated with LoF	Classification
10	8	41806856	*KAT6A*	ENST00000265713	c.1599-56_1621del	p.?	Splice acceptor variant	3	1.00	Developmental delay, language delay, hypotonia, specific facial features [45]	Pathogenic
13	18	42531084	*SETBP1*	ENST00000282030	c.1781del	p.P594Lfs*36	Frameshift	10	1.00	Developmental delay and language delay [46]	Pathogenic
	3	193380725	*OPA1*	ENST00000361908	c.2581C>T	p.R861*	Stop gained	10	0.99	Dominant optic atrophy	VUS
	1	21923758	*RAP1GAP*	ENST00000542643	c.*35-1G>A	p.?	Splice acceptor variant	18	0.97	NA	VUS
14	8	77761747	*ZFHX4*	ENST00000521891	c.3646-1G>A	p.?	Splice acceptor variant	1	1.00	Developmental delay and specific facial features (gene deletions only) [48]	Pathogenic
15	22	40662274	*TNRC6B*	ENST00000454349	c.2040G>A	p.W680*	Stop gained	3	1.00	Autism spectrum disorder [47]	Pathogenic
18	16	14234400	*MKL2*	ENST00000571589	c.-63-1G>C	p.?	Splice acceptor variant	3	1.00	NA	Likely pathogenic

Variants were classified according to recent guidelines [28]

*Chr* chromosome, *RVIS* residual variation intolerance score, *pLi* probability of being loss-of-function intolerant, *NA* not available, *VUS* variant of uncertain significance

A putative causal role for the multiple LoF mutations that we identified in the CAS cohort is further illustrated by comparison to WGS data from an independent control cohort of unaffected individuals, filtered in an identical manner (see “Patients and Methods” section). In these 22 unaffected controls, three putative LoF variants were identified in intolerant genes, none of which involved a gene previously identified as causal for a neurodevelopmental disorder (Supplementary Table 4).

Moreover, in independent exome sequencing of clinically-defined speech disorders, we identified an unrelated Australian case of CAS, together with oral apraxia, dysarthria, moderate ID, seizures and motor impairments, with a de novo LoF mutation disrupting *SETBP1*. This child carried a novel premature stop variant (c.C2665T, p.R889X) in *SETBP1* that was not present in her unaffected parents.

We also assessed rare missense and in-frame indel variants in our singleton CAS probands, again strictly filtered based on minor allele frequency, gene intolerance, predicted impact of the variant, and expression of the gene in developing brain (see “Methods” section). A total of 21 (0–4 per sample) rare, predicted damaging missense variants in intolerant genes were found (Supplementary Table 5). Again, variants were found in genes previously implicated in neurodevelopmental disorders. In particular, proband 19 carried a p.D2155N (c.6463G>A) variant in *TRIO*, a gene which has been associated with ASD and ID [50], and proband 18 carried a deletion of five amino acids (c.1029_1043del, p.Ala345_Ala349del) in *ARID1A*, one of the causal genes for Coffin–Siris syndrome [51]. When we analyzed WGS data from the 22 unaffected individuals of the control cohort using the same filtering steps, 17 rare missense mutations in intolerant genes were identified, of which six lay in a gene previously identified as causal for a neurodevelopmental disorder (Supplementary Table 4). Therefore, in absence of segregation information and functional evidence, we considered all the missense variants of the singleton CAS probands as variants of uncertain significance.

541 structural variants were detected in the WGS data of the singleton CAS probands, of which only one disrupted exonic regions of an intolerant gene. This 127-kb deletion (chr7:16,693,649–16,821,881), found in proband 14, disrupts all but the first exon of *BZW2* and the first five exons of *TSPAN13*. Neither gene has been associated with a neurodevelopmental disorder and no deletions of similar size at this location have been reported.

### A CAS-related co-expression module in developing brain tissue

Prior independent findings of protein-protein interactions between FOXP2 and CHD3 [52] and between SETD1A and WDR5 [43] imply that genes mutated in monogenic forms of CAS may belong to molecular networks with shared functionality. We formally tested this hypothesis with respect to the set of ten genes with strongest evidence for carrying causal variants in our whole-genome sequencing CAS analyses: *CHD3, SETD1A, WDR5, KAT6A, SETBP1, ZFHX4, TNRC6B, MKL2, ARID1A* and *TRIO*. These include the eight genes with a mutation that was classified as pathogenic or likely pathogenic, as well as the two genes that had a missense mutation and that were previously implicated in neurodevelopmental disorders.

An unbiased co-expression approach was taken to assess shared functionality. For this, we analyzed correlated patterns in gene expression levels in RNA-sequencing data of 224 samples from multiple brain regions of 23 human brains collected from 8 weeks post conception up to 1 year. Subcortical samples were included a priori, contrasting with a previous developmental brain co-expression analysis that utilized only cortical samples [37]. The inclusion of subcortical regions was based on a growing appreciation in the literature of their importance for speech and language development, including the identification of altered subcortical structure and function in individuals with language-related disorders [29], especially in CAS [30, 31]. Indeed, established CAS-related genes such as *FOXP2*, *BCL11A* and *ERC1* show high expression in subcortical tissues, and the effects of CAS mutations on basal ganglia function have been well documented [30–34].

**Fig. 2:**
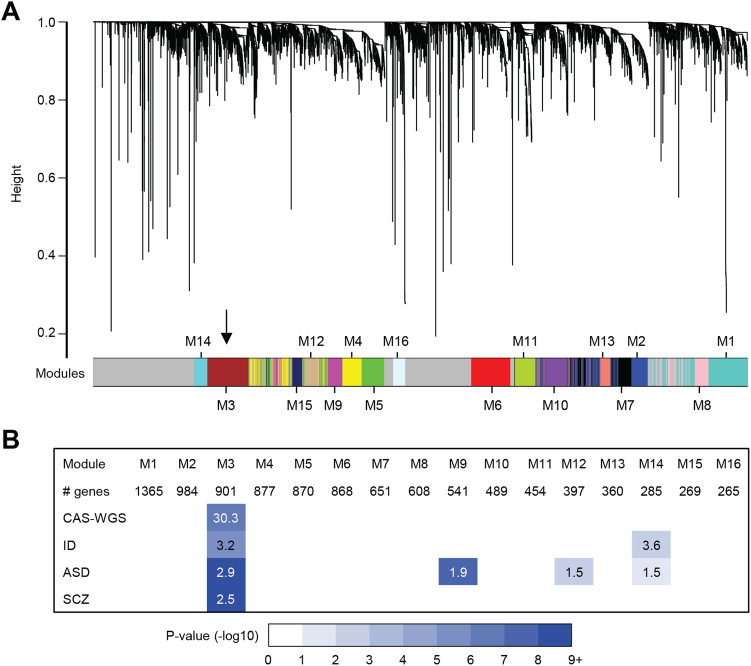
Co-expression network analysis. **a** A co-expression network was calculated using gene expression data of brain samples collected between 8 weeks post conception up to 1 year of age from the cortex, hippocampus, amygdala, striatum and thalamus. A total of 16 modules were detected. Module 3 (indicated by black arrow) was highly enriched for the genes we implicated in CAS through whole-genome sequencing (CAS-WGS). **b** Enrichment of developmental disorder gene sets in the 16 modules. Gene sets included are the 10 genes implicated in CAS through WGS (CAS-WGS), and genes with de novo mutations in patients with the following (1) intellectual disability (ID, *n* = 230), (2) autism spectrum disorder (ASD, *n* = 2760), and (3) schizophrenia (SCZ, *n* = 711). Significant enrichments with False discovery rate (FDR)-corrected *p* value < 0.05 and odds ratio (OR) >1 are shown. Colors indicate FDR-corrected *p* values for enrichment. Numbers show OR

A spatiotemporal co-expression network was calculated using weighted gene co-expression network analysis (WGCNA) [35] (Fig. 2a), yielding 16 co-expression modules, each comprising 265 to 1,365 genes. The genes that we had identified as potential risk factors in CAS were highly co-expressed in the developing human brain, with eight out of ten (all except *ZFHX4* and *MKL2*) belonging to a single shared module (M3 module; Fisher’s exact test: *p* = 1.09 × 10^−5^; FDR-corrected *p* = 1.85 × 10^−4^). This significant enrichment signal remained even if we excluded *ARID1A* and *TRIO* as genes with missense variants of uncertain significance (six out of eight genes belong to M3 module; FDR-corrected *p* = 4.96 × 10^−3^). Remarkably, although *FOXP2* could not be assigned to any module in this analysis, both *BCL11A* and *ERC1* were found to be members of this same module shared by the new CAS candidate genes. Genes carrying de novo mutations in ID, ASD and schizophrenia showed evidence of enrichment in module M3 as well (Fig. 2b). Genes implicated in ASD showed similar evidence for enrichment in modules M9, M12 and M14, and genes implicated in ID were enriched in module M14. By contrast, no enrichment was found for genes with LoF and predicted damaging missense mutations in intolerant genes identified in our WGS control cohort.

**Fig. 3:**
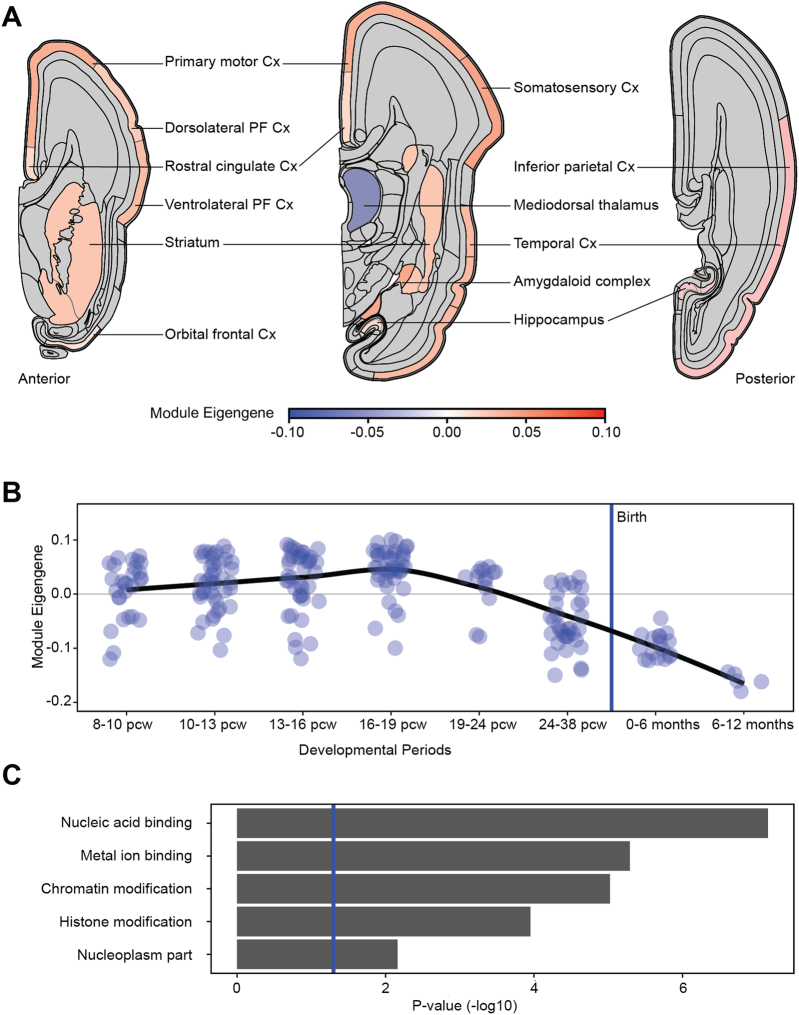
Human brain expression pattern and functional enrichment of module M3. **a** Spatial expression pattern of the CAS-related module at 13–24 weeks post conception, as visualized by the M3 module’s Eigengene. Red shows high expression, blue shows low expression. Multiple samples per region were averaged. No expression data were available for gray regions. **b** Developmental brain expression pattern of the enriched module during development, as visualized by the module Eigengene. Each dot represents a brain sample, the black line is the loess curve fitted through the data points. The blue vertical line represents time of birth. Pcw: post conception week. **c** Gene functions enriched in the module identified through gene ontology (GO) term enrichment followed by clustering of GO terms using the functional annotation clustering tool in DAVID. The *p* values represent the geometric mean of Bonferroni-corrected *p* values of all GO terms underlying each function. The blue vertical line represents the threshold for significant enrichment (*p* = 0.05). Cx cortex, PF prefrontal

Genes belonging to module M3 have high expression during early and mid-embryonic development in most brain regions, except for the mediodorsal nucleus of the thalamus, for which expression decreases during late fetal development and after birth (Fig. 3a, b). Analysis of biological pathways, using gene ontology terms, indicated that this CAS-related module is highly enriched for a number of classes, including genes involved in nucleic acid binding (726 genes, Bonferroni-corrected *p* = 7.05 × 10^−8^), which encompasses multiple transcription factors and genes involved in histone modification (400 genes, Bonferroni-corrected *p* = 1.12 × 10^−4^) (Fig. 3c). This finding is consistent with prior studies that implicated molecular pathways related to neurodevelopment and plasticity in speech disorders [33, 53], as well as in other neurodevelopmental disorders [54, 55]. ASD/ID-related modules M9 and M12 are also enriched for genes involved in transcription regulation, while ID-related module M14 is enriched for genes encoding synaptic components (Supplementary Fig. 2).

## Discussion

Over a decade and a half since identifying a role for the *FOXP2* gene in development of speech and language, we still have limited knowledge about other molecular factors that underlie these human capacities. Here we took advantage of advances in next-generation sequencing technologies as well as gene expression analyses of developing brain tissue, to reveal novel neurogenetic pathways implicated in speech development. Specifically, we applied whole-genome sequencing to a cohort of 19 probands ascertained on the basis of a rare severe developmental speech disorder with a distinct diagnosis, CAS. Based on prior studies of this rare phenotype, we hypothesized that CAS would be enriched for monogenic causes, involving disruptive mutations of large effect. In support of this hypothesis, we found that seven cases carried variants that were classified as pathogenic (in *CHD3*, *SETD1A*, *WDR5*, *KAT6A*, *SETBP1*, *TNRC6B* and *ZFHX4*) and another case carried a variant that was classified as likely pathogenic (in *MKL2*). These results strongly contrast with other more common language-related disorders that do not involve CAS. For example, a recent exome sequencing study on 43 unrelated probands with specific language impairment identified only a few possibly pathogenic mutations, and mainly supported a more complex pattern of inheritance, in which affected individuals carry multiple risk factors of modest effect size [56].

For half of the probands, whole-genome sequence data from both parents allowed for the isolation of de novo mutations, implicating the genes *CHD3*, *SETD1A* and *WDR5* in the pathogenesis of CAS for the first time. Remarkably, a previous systematic yeast-two-hybrid screen for interaction partners of FOXP2, the most well-established CAS-related gene, identified the CHD3 protein as one of the top putative interactors of this transcription factor (see Table S1 in reference [52]). Our findings are consistent with the identification of multiple de novo mutations disrupting the same functional domain of *CHD3* in other patients with neurodevelopmental disorders involving speech and language problems (L. Snijders Blok, personal communication). Beyond *CHD3*, another seven of the genes highlighted by our WGS analyses (*SETD1A, KAT6A, SETBP1, TNRC6B, ZHFX4, ARID1A* and *TRIO*) have been linked to related neurodevelopmental phenotypes in prior published work. Moreover, for a number of these genes, including *SETD1A*, *KAT6A*, *SETBP1* and *TRIO*, the associated disorders, while being broader and/or more severe than CAS, have nevertheless been noted to include speech and language deficits. A particularly interesting example is *SETBP1*, haploinsufficiency of which has been previously associated with a phenotype including mild to severe ID, motor delay, facial dysmorphism and expressive language delay [46]. In the present study, we identified a *SETBP1* frameshift mutation in our WGS cohort. A de novo LoF variant disrupting *SETBP1* in an additional case with CAS, which we identified through independent exome sequencing, confirmed the relevance of this gene for developmental speech deficits. In addition, in a recent genome-wide screen of a geographically isolated Russian population with high prevalence of developmental language disorder, common variants of *SETBP1* were significantly associated with complexity of linguistic output [57].

It is well established that diverse neurodevelopmental consequences can result from mutations in the same gene [58], hence it is perhaps not surprising that genes involved in other neurodevelopmental phenotypes may be implicated in primary speech disorders [8–10]. This holds even for *FOXP2*, the most-well studied gene in the language sciences, mutations of which were recently shown to cause a range of phenotypic profiles in different cases: various types of speech and language impairment with or without mild cognitive impairment or mild delays in motor development [59]. Our findings are consistent with this picture—while CAS was the major feature used to diagnose probands in the present study, some of the affected children also showed signs of reduced cognitive function and/or deficits in motor development. CAS may therefore be considered as a part of a range of neurodevelopmental brain disorders with a shared genetic foundation, instead of a pure isolated phenotype involving genes that are exclusively related to speech disorders.

Using expression data from multiple regions of the developing human brain, we discovered that the genes identified in our CAS whole-genome screening have highly correlated gene expression patterns; eight out of ten (all but *ZHFX4* and *MKL2*) are members of a single co-expression module, including mostly transcription factors and chromatin remodelers with high expression during early and mid-fetal brain development. This finding is in line with the known molecular functions of the genes that we identified through WGS, since most are directly involved in regulating gene expression, and are functionally connected with each other. *CHD3* encodes a chromatin remodeler that is part of a protein complex (the NuRD complex) regulating gene repression [60], and both *SETBP1* and *ZHFX4* encode transcription factors that can interact with the NuRD complex [61, 62]. As noted above, FOXP2 interacts with CHD3 [52], providing a direct link between the novel genes and earlier established pathways in CAS. *SETD1A* and *WDR5* encode different parts of a protein histone methyltransferase complex named SET1/MLL [43]. *KAT6A* codes for the histone-acetyl-transferase of the MOZ complex that establishes gene activation, also through interaction with SET1/MLL [63]. The protein encoded by *ARID1A* is part of a large chromatin remodeling complex (called SNF/SWI) [64], and *MKL2* encodes a subunit of the stimulus-dependent transcription factor SRF [65]. Finally, *TNRC6B* is involved in micro-RNA-directed RNA processing [66], playing a more indirect role in gene expression regulation.

The enrichment of CAS-associated genes in the co-expression module suggests that regulation of gene expression during early human brain development plays a role in susceptibility to this disorder. Genes implicated in ID, ASD and schizophrenia are also enriched in module M3, in line with previous reports highlighting the involvement of chromatin remodelers and transcription factors in these neurodevelopmental disorders [37, 54, 55]. However, the degree of enrichment is much lower than that observed for the CAS candidate genes. In addition, genes related to ID and ASD show enrichment in the synaptic gene module M14, which is consistent with previous reports linking synaptic gene function to autistic phenotypes [37, 54]. Thus, we propose that a subset of genes involved in neurodevelopmental disorders—transcription factors and chromatin remodelers mostly expressed during early brain development—are particularly relevant for speech development. Dynamic chromatin-level modifications of the genome are crucial for coordination of multiple stages of neural development [67]. For example, several of the CAS-related proteins in module M3 belong to chromatin remodeling and/or transcription factor complexes that have been shown to play key roles in neuronal differentiation and cortical layer specification in mouse models [68, 69].

Given the well-established involvement of cortical and subcortical brain structures in pathology of CAS [30, 31], we calculated the co-expression network for this study based on both cortical and subcortical tissues. As a post hoc analysis, we compared our network findings to a network from a prior ASD investigation that used the same primary source of RNA data, but limited to only cortical samples [37] (Supplementary Table 6). While the inclusion or exclusion of subcortical structures obviously yields differences between the derived networks of the two studies, the clustering of our CAS candidates is robust. Five genes (*CHD3*, *SETD1A*, *SETBP1*, *ARID1A* and *TRIO*) are part of module M2 from the cortical-only study and the enrichment is significant (FDR-corrected *p* value 2.0 × 10^−4^). Module M2 (containing 1,036 genes) is the closest match in the cortical-only network to module M3 (containing 901 genes) of our cortical-subcortical network, with 295 overlapping genes. These modules show similar developmental expression trajectories and GO term enrichments.

Similarity in function and developmental brain expression pattern provided support for a causal relation between the variants we identified through WGS and speech deficits. However, interpretation of WGS results is challenging in cases when recurrent mutations cannot provide final proof for a causal relation between the identified genes and disorder [70]. Our report is the first to associate heterozygous variants of *WDR5* with a neurodevelopmental disorder, therefore further evidence of causative variants in children with CAS or a related phenotype will be important to confidently implicate this gene. *ARID1A* and *TRIO* were already associated with neurodevelopmental disorder, and the missense mutations in these genes were selected as potential causal variants in proband 18 and 19, respectively. Disease-causing missense variants in *TRIO* have so far been described mostly in the first Dbl homology-Pleckstrin homology (DH-PH) domain [50], while the p.D2155N variant identified in case 19 is located adjacent to the second DH-PH domain. In addition, mostly LoF mutations have been described in *ARID1A*, and the impact of missense mutations is difficult to predict without functional studies. Analyses of our control cohort suggested that the strict filtering criteria might have increased the chance of finding missense mutations in genes associated with neurodevelopmental disorders. Therefore, we treat these two genes with some degree of caution at this point. Our WGS analysis identified another 19 missense variants that were predicted as damaging and located in intolerant genes. Despite very strict filtering criteria, we cannot infer causality for such variants without additional cases. For example, in *HERC1*, recurrent missense mutations were identified: a de novo p.S3485N variant in proband 08, and a p.E978S variant in proband 15. To date, only recessive mutations in *HERC1* have been found as causal for intellectual disability [44], and no phenotype has been described for the parents harboring a single *HERC1* variant, so the relevance of *HERC1* missense variants for CAS cannot currently be determined. Lastly, we cannot exclude a more complex pattern of inheritance in the probands who carry more than one variant that was predicted to be damaging.

In summary, by analyzing whole-genome sequences from 19 probands ascertained through a CAS diagnosis, we identified de novo mutations in *CHD3, SETD1A* and *WDR5* and LoF mutations in *SETBP1*, *KAT6A*, *TNRC6B* and *ZFHX4* that were all classified as pathogenic. Moreover, we implicated a network of functionally connected genes, part of a coordinated expression module in the embryonic human brain, in the development of proficient speech skills. A significant number of these genes (*CHD3, SETD1A, KAT6A, SETBP1, TNRC6B, ZFHX4, ARID1A* and *TRIO*) have been associated with neurodevelopmental disorders with or without speech problems [42, 45–51]. These results indicate that the genetic underpinnings of CAS are—at least in part—shared with those of other brain-related syndromes, but that genes relevant for speech are clustered within particular functional networks. Our work identifies molecular pathways involved in regulation of gene expression during early brain development that may be critical for the acquisition of fluent spoken language.

## Electronic supplementary material

Supplementary Information
