# Supplementary material for: A set of regulatory genes co-expressed in embryonic human brain is implicated in disrupted speech development
Source: Mol Psychiatry. 2018 Feb 20;24(7):1065–78. doi: 10.1038/s41380-018-0020-x (PMC6756287; doi:10.1038/s41380-018-0020-x)
Supplement: Supplementary file 1 — Supplementary Information [file 41380_2018_20_MOESM1_ESM.pdf]

Supplementary Figures

Module	M1	M2	M3	M4	M5	M6	M7	M8	M9	M10	M11	M12	M13	M14	M15	M16
# genes	1365	984	901	877	870	868	651	608	541	489	454	397	360	285	269	265
Intolerant			4.3	1.3	1.4				3.3			3.2		3.1		
Intolerant to LoF			4.4	1.3	1.5				3.5			3.3		2.9		
Intolerant to missense	1.4		3.5						2.7			2.4		2.9		

P-value (-log10) 0 1 2 3 4 5 6 7 8 9+

Figure S1: Enrichment of sets of intolerant genes in the 16 modules. Gene sets included are 1) the 2143 genes intolerant to loss-of-function and/or missense mutations, as indicated by a pLi score > 0.9 and/or a mis_z score >3, 2) the 1919 genes intolerant to loss-of-function mutations, as indicated by a pLi score > 0.9 and 3) the 1035 genes intolerant to missense mutations as indicated by a mis_z score > 3. Significant enrichments with False discovery rate (FDR)-corrected p-value <0.05 and odds ratio (OR) >1 are shown. Colours indicate FDR-corrected p-values for enrichment. Numbers show OR.

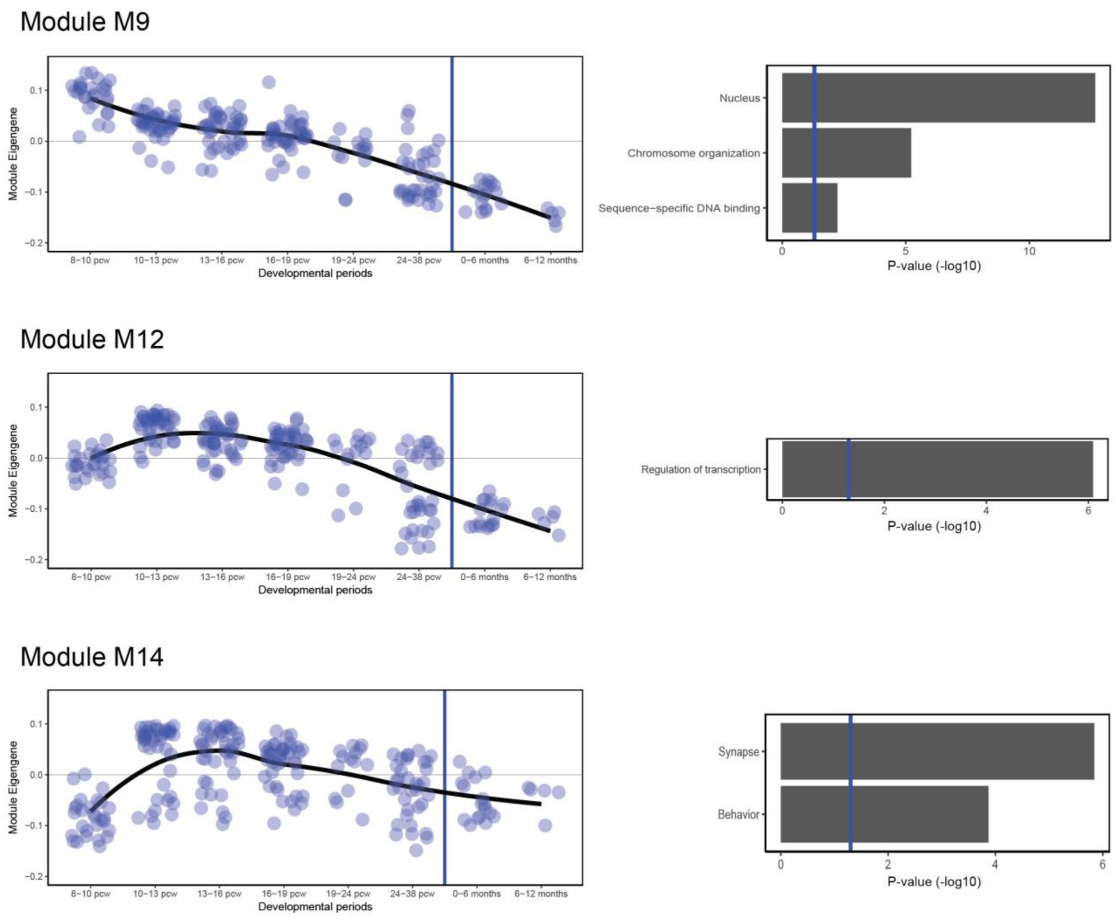

Figure S2: Human brain expression pattern and functional enrichment of module M9, M12 and M13. Left panels show the developmental brain expression pattern of the module during development, as visualized by the module Eigengene. Each dot represents a brain sample, the black line is the loess curve fitted through the data points. The blue vertical line represents time of birth. Pcw: post conception week. Right panels show the gene functions enriched in the modules identified through gene ontology (GO) term enrichment followed by clustering of GO terms using the functional annotation clustering tool in DAVID. The p-values represent the geometric mean of Bonferroni-corrected p-values of all GO terms underlying each function. The blue vertical line represents the threshold for significant enrichment ($p = 0.05$).

Supplementary Tables

Table S1: Phenotype data.

Proband	Gender	Age (years)	CAS	Age at beginning treatment (years)	Late onset language use[a]	Listening Comprehension Scales standard scores <85	Description IQ[b]	Oral Expression Scales standard scores <85	Gross or fine motor impairment[c]	Oral nonverbal motor impairment[d]	Dysarthria
01	M	9	+	0.5	+	NA	Below average	NA	+	+	NA
02	F	16	+	NA	+	+	Lower extreme	+	+	NA	+
03	F	7	+	NA	NA		Average		+	+	
04	F	6	+	NA	+	+	Average	+	+	+	+
05	F	19	+	NA	+	+	NA	+	NA	NA	
06	M	5	+	1.5	+		NA	+	+	NA	NA
07	F	7	+	7	+	+	Lower extreme	+	+	NA	+
08	F	3	+	NA	+	+	NA	+	+	NA	NA
09	M	4	+	3	+	+	NA	+		NA	
10	M	11	+	5.5	+	+	Average	+		+	+
11	M	10	+	4	+		Average		+	+	+
12	M	6	+	1	+	+	Average	+	+	+	
13	F	12	+	4	+	+	Lower extreme	+	+		+
14	M	7	+	3	+		Average		+	+	+
15	F	4	+	NA	+		Average		+	+	
16	M	4	+	2	+		Average		+		
17	F	7	+	2	+	+	Below average	+	+		+
18	F	12	+	1.5	+	+	Lower extreme	+	+	+	+
19	F	6	+	2	+		NA	+	+		NA

Plus-sign ('+') indicates impairment, blank cells indicate negative history or performance within normal limits. [a]Based on parent report of late onset of language use. [b]Below average: IQ between 84 and 70; Lower extreme: IQ below 70. [c]Based on parent report or history of physical or occupational therapy. [d]Based on oral-nonverbal motor assessment task. NA: not available.

Table S2: Whole genome sequencing CAS cohort and overlap with previously published cohorts.

Family	Sample	Worthey et al. 2013 proband ID	Laffin et al. 2012 proband ID	Family member	Genetic variation reported by Worthey et al. 2013 and Laffin et al. 2012
01	Mother 01			Mother	
01	Father 01			Father	
01	Proband 01	Patient 8		**Proband**	*FOXP1* I107T
02	Mother 02			Mother	
02	Father 02			Father	
02	Proband 02	Patient 5		**Proband**	Nothing reported
03	Mother 03			Mother	
03	Father 03			Father	
03	Proband 03	Patient 7		**Proband**	*CNTNAP1* R1064Q
04	Mother 04			Mother	
04	Father 04			Father	
04	Proband 04	Patient 6		**Proband**	*CNTNAP2* R171C
05	Mother 05			Mother	
05	Father 05			Father	
05	Proband 05	Patient 3		**Proband**	Nothing reported
06	Mother 06			Mother	
06	Father 06			Father	
06	Proband 06	Patient 2	Patient 3	**Proband**	1.8Mb deletion at 2q31.1 & *ATP13A4* E646D
06	Sibling 06			Sibling (M)	
07	Mother 07			Mother	
07	Father 07			Father	
07	Proband 07			**Proband**	
08	Mother 08			Mother	
08	Father 08			Father	
08	Proband 08		Patient 1	**Proband**	127kb deletion at 8q21.13
09	Mother 09			Mother	

09	Father 09			Father	
09	Proband 09		Patient 2	**Proband**	0.7Mb duplication at 6p12.1
10	Proband 10	Patient 4		**Proband**	*CNTNAP2* splice consensus deletion & *ATP13A4* E646D
11	Proband 11		Patient 8	**Proband**	0.2Mb deletion at 2q31, *FOXP2* N622H
12	Proband 12			**Proband**	
13	Proband 13		Patient 11	**Proband**	90kb duplication at 4p15.1 & 53kb deletion at 17q23.2
14	Proband 14			**Proband**	
15	Proband 15			**Proband**	
16	Proband 16			**Proband**	
17	Proband 17			**Proband**	
18	Proband 18			**Proband**	
19	Proband 19			**Proband**	

Family structure of the dataset, and information on how the samples of the present study correspond to the previously published whole exome sequencing analysis by Worthey et al. 2013[1] and array comparative genomic hybridization analysis by Laffin et al. 2012.[2]

Table S3: *De novo* exonic protein-altering variants in CAS trios.

Proband	Chr	Base	Gene	Transcript	cDNA change	Protein change	Impact	RVIS	pLi	MIS_Z	GERP	CADD	Polyphen[a]	Sift[b]	Classification
01	17	7806599	*CHD3*	ENST00000380358	c.3682C>T	p.R1228W	Missense	1	1.00	7.15	1.1	15	1.00	0.00	Pathogenic
02	18	9221974	*ANKRD12*	ENST00000262126	c.920C>G	p.S307C	Missense	2	1.00	-1.23	5.6	15	0.99	0.00	VUS
	11	62594638	*STX5*	ENST00000294179	c.412A>G	p.I138V	Missense	64	0.36	0.56	5.5	17	0.94	0.38	VUS
	19	607997	*HCN2*	ENST00000251287	c.1252C>G	p.L418V	Missense	2	0.83	7.27	3.1	17	0.47	0.16	VUS
04	16	30976714	*SETD1A*	ENST00000262519	c.1652_1656dup	p.P553Wfs*110	Frameshift	1	1.00	2.91	5.5	NA	NA	NA	Pathogenic
07	9	137017143	*WDR5*	ENST00000358625	c.623C>T	p.T208M	Missense	21	1.00	3.45	3.7	21	0.59	0.00	Pathogenic
08	1	212792854	*ATF3*	ENST00000366981	c.503A>C	p.N168T	Missense	63	0.28	1.18	5.0	17	0.24	0.00	VUS
	8	144944141	*EPPK1*	ENST00000525985	c.3281G>A	p.S1094N	Missense	NA	0.00	-2.73	3.4	11	0.05	0.01	VUS
	15	63943544	*HERC1*	ENST00000443617	c.10454G>A	p.S3485N	Missense	0	1.00	3.83	5.4	17	0.02	0.65	VUS

Variants were classified according to recent guidelines. Interpretation of the scores can be found in Table 2. Chr: chromosome, RVIS: Residual Variation Intolerance Score, pLi: probability of being loss-of-function intolerant, MIS_Z: Z-score for missense constraint, GERP: Genomic Evolutionary Rate Profiling, CADD: Combined Annotation Dependent Depletion, Polyphen: Polymorphism Phenotyping, Sift: Sorting Intolerant from Tolerant, NA: not available.

Table S4: Rare LoF and high-impact missense variants in intolerant genes in healthy control cohort.

Control	Chr	Base	Gene	Transcript	cDNA change	Protein change	Impact	RVIS	MIS_Z	pLi	GERP	CADD	Polyphen[a]	Sift[b]	Phenotype previously associated with heterozygous variant
3	1	32503580	KHDRBS1	ENST00000327300	c.1049C>T	p.A350V	Missense	18	3.50	NA	5.8	33	PosD	D	NA
	6	157222592	ARID1B	ENST00000275248	c.1645C>G	p.P549A	Missense	2	3.39	NA	3.5	16	PrD	NA	Coffin-Siris syndrome
4	19	17728526	UNC13A	ENST00000252773	c.4544C>T	p.T1515M	Missense	2	5.89	NA	4.0	16	PosD	D	Dyskinetic movement disorder, developmental delay and autism
5	15	30010626	TJP1	ENST00000346128	c.3718_3719del	p.N1240fs	Frameshift	4	NA	1.00	NA	NA	NA	NA	NA
	18	12311020	TUBB6	ENST00000317702	c.244G>A	p.G82R	Missense	15	4.11	NA	4.8	18	PrD	D	Congenital non-progressive bilateral facial palsy and congenital velopharyngeal dysfunction
6	11	61632678	FADS2	ENST00000257261	c.1147_1148del	p.S383fs	Frameshift	11	NA	0.99	NA	NA	NA	NA	NA
	18	3879285	DLGAP1	ENST00000315677	c.785G>A	p.C262Y	Missense	2	4.01	NA	5.5	23	PosD	D	NA
7	3	51746679	GRM2	ENST00000395052	c.640G>A	p.G214S	Missense	18	3.27	NA	5.0	32	PrD	D	NA
8	4	134084252	PCDH10	ENST00000264360	c.2917C>A	p.L973M	Missense	2	4.47	NA	5.2	25	PrD	D	NA
	5	147023758	JAKMIP2	ENST00000265272	c.1088A>G	p.E363G	Missense	10	3.82	NA	5.5	23	PosD	D	NA
9	9	96320927	FAM120A	ENST00000277165	c.2732G>A	p.R911H	Missense	2	4.63	NA	5.6	27	PosD	D	NA
10	9	130438999	STXBP1	ENST00000373299	c.1325A>G	p.N442S	Missense	15	5.22	NA	5.2	25	PosD	D	Epileptic encephalopathy, early infantile, 4
	11	105483154	GRIA4	ENST00000282499	c.238C>T	p.T80I	Missense	3	3.16	NA	5.7	27	PrD	D	NA
11	6	3850147	FAM50B	ENST00000380272	c.101G>A	p.R34H	Missense	25	3.41	NA	3.3	21	PosD	D	NA
	11	10781814	CTR9	ENST00000361367	c.686C>G	p.S229C	Missense	6	4.44	NA	4.8	22	PosD	D	NA
14	8	48765330	PRKDC	ENST00000314191	c.6908G>A	p.V2303*	Stop gained	NA	NA	1.00	NA	NA	NA	NA	NA
16	19	4552471	SEMA6B	ENST00000301293	c.953C>T	p.P318L	Missense	9	3.41	NA	4.1	20	PrD	D	NA
17	1	8555212	RERE	ENST00000337907	c.1016C>T	p.A339V	Missense	1	3.57	NA	5.8	23	PrD	NA	Neurodevelopmental disorder with or without anomalies of the brain, eye, or heart
22	17	65928064	BPTF	ENST00000306378	c.6187G>A	p.V2063M	Missense	0	4.39	NA	5.5	18	PrD	NA	Syndromic developmental and speech delay, postnatal microcephaly and dysmorphic features.
	3	48691791	CELSR3	ENST00000164024	c.5084G>A	p.R1695Q	Missense	4	6.17	NA	3.9	28	PosD	D	NA

Chr: chromosome, RVIS: Residual Variation Intolerance Score, MIS_Z: Z-score for missense constraint, GERP: Genomic Evolutionary Rate Profiling, CADD: Combined Annotation Dependent Depletion, Polyphen: Polymorphism Phenotyping, Sift: Sorting Intolerant from Tolerant, NA: not available or not applicable. [a]PrD: probably damaging, PosD: possibly damaging. [b]D: deleterious.

Table S5: Rare high-impact missense variants in intolerant genes in CAS singletons.

Proband	Chr	Base	Gene	Transcript	cDNA change	Protein change	RVIS	MIS_Z	GERP	CADD	Polyphen[a]	Sift[b]	Phenotype previously associated with heterozygous missense variants
11	1	19493579	*UBR4*	ENST00000375254	c.4046G>C	R1349P	0	5.98	5.8	18	PrD	D	NA
12	12	117669852	*NOS1*	ENST00000317775	c.3320C>T	p.T1107M	5	3.82	4.6	23	PrD	D	NA
	19	42858839	*MEGF8*	ENST00000251268	c.4111C>T	p.R1371W	4	3.28	3.2	18	PrD	D	NA
13	19	38980762	*RYR1*	ENST00000355481	c.5861G>A	p.R1954H	0	4.44	4.6	21	PosD	D	Central core disease, multiminicore disease and malignant hyperthermia
	5	74650932	*HMGCR*	ENST00000287936	c.1615G>A	p.G539R	7	4.05	6.1	35	PrD	D	NA
	2	128767945	*SAP130*	ENST00000357702	c.845C>G	p.S282C	2	3.11	5.6	21	NA	D	NA
14	11	117667871	*DSCAML1*	ENST00000321322	c.104G>A	p.R35H	0	4.25	4.6	21	NA	NA	NA
15	19	7132235	*INSR*	ENST00000302850	c.2776C>T	p.R926W	4	5.21	2.5	20	PrD	D	Insulin-resistant diabetes mellitus and acanthosis nigricans
	15	64021785	*HERC1*	ENST00000443617	c.2932_2933GA>TC	p.E978S	0	3.83	5.3	NA	PosD	D	NA
	22	46929632	*CELSR1*	ENST00000262738	c.3436A>G	p.N1146D	2	4.42	3.6	18	PrD	D	Spina bifida, craniorachischisis
	19	41114185	*LTBP4*	ENST00000396819	c.1216C>T	p.R406C	NA	3.23	4.0	20	PrD	D	NA
16	20	3147638	*LZTS3*	ENST00000329152	c.157C>T	p.R58C	12	3.15	4.9	21	PosD	D	NA
17	12	94543243	*PLXNC1*	ENST00000258526	c.496G>A	p.V166M	3	4.76	4.7	17	PrD	D	NA
	7	127341233	*SND1*	ENST00000354725	c.445C>T	p.L149F	6	3.35	6.2	30	PrD	D	NA
	17	40068741	*ACLY*	ENST00000352035	c.214G>T	p.V72F	2	3.28	4.8	25	PrD	D	NA
	11	105850372	*GRIA4*	ENST00000282499	c.2615G>A	p.R872H	3	3.16	5.6	27	PrD	D	NA
18	1	27023908	*ARID1A*	ENST00000324856	c.1029_1043del	p.Ala345_Ala349del	1	4.10	3.8	NA	NA	NA	Coffin-Siris syndrome
19	5	14481725	*TRIO*	ENST00000537187	c.6463G>A	p.D2155N	0	6.29	5.3	33	PrD	D	Mild intellectual disability
	3	48684209	*CELSR3*	ENST00000164024	c.7282C>T	p.R2428C	4	6.17	5.8	18	PrD	D	NA
	15	73659860	*HCN4*	ENST00000261917	c.752G>A	p.G251E	7	4.83	4.8	21	NA	D	Sinus bradycardia and other heart arrhythmias
	1	32201968	*BAI2*	ENST00000373658	c.3154G>A	p.V1052M	6	4.82	4.7	19	PrD	D	NA

Chr: chromosome, RVIS: Residual Variation Intolerance Score, MIS_Z: Z-score for missense constraint, GERP: Genomic Evolutionary Rate Profiling, CADD: Combined Annotation Dependent Depletion, Polyphen: Polymorphism Phenotyping, Sift: Sorting Intolerant from Tolerant, NA: not available. [a]PrD: probably damaging, PosD: possibly damaging. [b]D: deleterious.

Table S6: Number of genes overlapping between modules of co-expression networks from Parkishak (cortical-only) and Eising (cortical-subcortical) studies.

	Parikshak *et al.* modules																
Eising *et al.* modules	**M1**	**M2**	**M3**	**M4**	**M5**	**M6**	**M8**	**M9**	**M10**	**M11**	**M12**	**M13**	**M14**	**M15**	**M16**	**M17**	**M18**
**M1**	-	-	-	52	1	-	-	5	-	-	4	171	-	11	125	369	6
**M2**	271	-	-	21	-	-	41	1	-	23	4	54	-	39	-	12	127
**M3**	-	295	66	-	78	93	-	-	-	1	5	-	2	-	2	1	-
**M4**	2	7	226	4	1	7	-	14	-	32	-	-	154	-	-	-	-
**M5**	-	43	113	1	218	1	-	15	-	-	-	-	71	-	18	1	-
**M6**	12	20	1	-	1	97	1	4	-	3	279	8	4	4	-	7	5
**M7**	126	-	8	2	-	2	124	-	-	106	6	5	3	5	-	-	14
**M8**	16	-	-	32	-	-	-	2	-	-	-	151	-	22	1	109	9
**M9**	1	57	206	-	9	10	-	-	-	8	2	-	5	-	-	-	-
**M10**	5	-	-	-	-	-	-	-	-	1	-	21	-	6	-	7	322
**M11**	25	-	16	-	-	1	19	2	-	240	-	-	14	1	-	-	-
**M12**	-	79	22	-	114	8	-	-	-	-	-	-	3	-	12	-	-
**M13**	96	-	-	8	-	-	2	-	-	1	-	52	-	24	-	17	24
**M14**	-	23	10	-	71	-	-	-	-	-	-	-	2	-	21	11	-
**M15**	-	-	-	12	5	2	-	29	-	-	-	-	31	-	15	3	-
**M16**	-	-	-	1	-	-	-	2	-	-	-	14	-	-	126	28	1

Numbers represent total overlapping genes between two modules of the co-expression network published by Parikshak *et al.*[3] and the co-expression network described here. Genes not assigned to any module are not included in the table.

Supplementary References

1. Worthey EA, Raca G, Laffin JJ, Wilk BM, Harris JM, Jakielski KJ*, et al.* Whole-exome sequencing supports genetic heterogeneity in childhood apraxia of speech. *Journal of neurodevelopmental disorders* 2013, **5**(1)**:** 29.

2. Laffin JJ, Raca G, Jackson CA, Strand EA, Jakielski KJ, Shriberg LD. Novel candidate genes and regions for childhood apraxia of speech identified by array comparative genomic hybridization. *Genetics in medicine : official journal of the American College of Medical Genetics* 2012, **14**(11)**:** 928-936.

3. Parikshak NN, Luo R, Zhang A, Won H, Lowe JK, Chandran V*, et al.* Integrative functional genomic analyses implicate specific molecular pathways and circuits in autism. *Cell* 2013, **155**(5)**:** 1008-1021.
